# Clinical practice of non-invasive ventilation for acute exacerbations of chronic obstructive pulmonary disease

**DOI:** 10.1186/s12931-023-02507-1

**Published:** 2023-08-23

**Authors:** Judith Elshof, Judith M. Vonk, Anouschka van der Pouw, Cella van Dijk, Petra Vos, Huib A.M. Kerstjens, Peter J. Wijkstra, Marieke L. Duiverman

**Affiliations:** 1 Department of Pulmonary Diseases/Home Mechanical Ventilation, University of Groningen, University Medical Center Groningen, Groningen, The Netherlands; 2 Groningen Research Institute for Asthma and COPD (GRIAC), University of Groningen, University Medical Center Groningen, Groningen, The Netherlands; 3 Department of Epidemiology, University of Groningen, University Medical Center Groningen, Groningen, The Netherlands; 4 Department of Pulmonary Diseases, Rijnstate Hospital, Arnhem, The Netherlands

**Keywords:** COPD, Exacerbation, Acute respiratory failure, Non-invasive ventilation

## Abstract

**Background:**

Non-invasive ventilation (NIV) is an evidence-based treatment for acute respiratory failure in chronic obstructive pulmonary disease (COPD). However, suboptimal application of NIV in clinical practice, possibly due to poor guideline adherence, can impact patient outcomes. This study aims to evaluate guideline adherence to NIV for acute COPD exacerbations and explore its impact on mortality.

**Methods:**

This retrospective study was performed in two Dutch medical centers from 2019 to 2021. All patients admitted to the pulmonary ward or intensive care unit with a COPD exacerbation were included. An indication for NIV was considered in the event of a respiratory acidosis.

**Results:**

A total of 1162 admissions (668 unique patients) were included. NIV was started in 154 of the 204 admissions (76%) where NIV was indicated upon admission. Among 78 admissions where patients deteriorated later on, NIV was started in 51 admissions (65%). Considering patients not receiving NIV due to contra-indications or patient refusal, the overall guideline adherence rate was 82%. Common reasons for not starting NIV when indicated included no perceived signs of respiratory distress, opting for comfort care only, and choosing a watchful waiting approach. Better survival was observed in patients who received NIV when indicated compared to those who did not.

**Conclusions:**

The adherence to guidelines regarding NIV initiation is good. Nevertheless, further improving NIV treatment in clinical practice could be achieved through training healthcare professionals to increase awareness and reduce reluctance in utilizing NIV. By addressing these factors, patient outcomes may be further enhanced.

**Supplementary Information:**

The online version contains supplementary material available at 10.1186/s12931-023-02507-1.

## Introduction

Non-invasive ventilation (NIV) is an evidence-based treatment for patients with acute respiratory failure due to an exacerbation of Chronic Obstructive Pulmonary Disease (COPD). In COPD patients with acute hypercapnic respiratory failure, NIV improves gas exchange, reduces work of breathing and reduces length of hospital stay and mortality [1, 2]. Furthermore, when compared to invasive ventilation, NIV leads to fewer complications, such as ventilator related infections [3, 4]. These findings have resulted in guideline recommendations for the use of NIV in acute respiratory failure due to an exacerbation of COPD [5].

However, the real effectiveness of NIV in routine clinical practice is uncertain. Kaul et al. [6] performed a large observational multicenter study including 7529 COPD patients with an exacerbation. They found a higher in-hospital mortality rate among patients who received NIV in comparison to those who received conventional care, which contradicts the results of earlier performed randomized trials on which the guidelines regarding NIV are based. The same research group performed a follow-up study with 9716 patients to provide possible explanations for their relatively high mortality [7]. They suggested that this discrepancy might be explained by the fact that patients in clinical practice are more severely acidotic than patients included in the randomized trials. Another explanation for the difference in mortality rate might be that the guideline regarding NIV initiation is often not followed. The guideline states a clear indication for NIV during an exacerbation of COPD: moderate to severe respiratory acidosis in the arterial blood gas analysis (i.e. pH < 7.35 and partial pressure of carbon dioxide (PaCO_2_) > 6.0 kPa) without contraindications. Despite this explicit indication, Roberts et al. [7] reported that both the initiation of NIV in patients with a metabolic acidosis and the non-initiation in patients with a respiratory acidosis were no exception. This is in agreement with another study by Roberts et al. [8], which showed that adherence to the guidelines concerning COPD exacerbations in general, and also specifically to NIV treatment during COPD exacerbations, is poor. They reported that only 51% of the patients who fulfilled the indications for NIV received NIV. Vice versa, they describe that 29% of all patients receiving NIV did not fulfil the criteria for NIV. Overall, earlier research suggests that the application of NIV is far from optimal in daily clinical practice, which may have detrimental effects on patient outcomes. However, due to the large cohorts in these studies, it was impossible to state the rationale behind the (non-)initiation of NIV and whether it was applied correctly at case-level, making it difficult to draw conclusions about the adherence to NIV guidelines. Furthermore, we wondered whether there could be relevant differences between countries, in this case, between the United Kingdom and the Netherlands.

Therefore, the goal of this study is to describe adherence to guidelines concerning NIV for acute COPD exacerbations in two medical centers in the Netherlands, and investigate its effect on mortality.

## Methods

### Study design

This retrospective study was performed in the departments of pulmonary diseases in two medical centers in the Netherlands: hospital A, an academic hospital, where screening took place between January 2019 and July 2021, and hospital B, a large non-university teaching hospital, where screening was conducted between April 2019 and January 2021.

Patients had to meet the following criteria to be included: a history of COPD and admission to the hospital with an exacerbation of COPD. History of COPD was based on prior pulmonary function tests, either performed in hospital or at the general practitioner’s practice. An exacerbation was defined as a period of worsening of symptoms treated with oral prednisolone and/or antibiotics. The only exclusion criterium was admittance to a department other than the pulmonary department or the intensive care unit.

The medical ethics committee of the University Medical Center Groningen examined the research protocol and decided that the study was not subject to the Dutch Research on Humans Subjects Act and waived the need for formal ethics approval and informed consent. However, to comply with local regulations, all living study participants were requested to declare any objection for data usage and absence of objection was regarded as their consent.

### Data collection

One researcher collected data by analysing medical records that had already been gathered as part of standard clinical care. The following data were obtained and entered into a database (SPSS Inc., Chicago, Ill, USA): demographic characteristics, medical history, lab results, admission and treatment details, and mortality (in-hospital and 90 days after hospital discharge).

An indication for NIV was considered in the event of a respiratory acidosis (pH < 7.35 and pCO_2_ > 6.0 kPa) detected by the arterial or capillary blood gas analysis. The origin of the exacerbation was classified as infectious based on a positive bacterial culture or nasopharyngeal viral swab, or a high clinical suspicion of infection based on the patient’s symptoms, high level of C-reactive protein, and/or the identification of an infiltrate on chest radiograph. If there was an obvious non-infectious origin, such as exposure to irritants or neglecting the use of inhalation medication, the exacerbation was classified as non-infectious. In cases where neither of these origins applied, the origin was marked as unknown. The classification of co-morbidities involved a review of both medical history and medication use. The NIV protocol used at both hospitals is presented in the Supplementary Materials.

### Statistics

Descriptive analyses were used to calculate adherence to guidelines. Results are given as median with interquartile range. Differences in numerical data were analysed using an unpaired t-test or Mann-Whitney U test, depending on their distribution. Comparison of categorical data were analysed by using Fisher’s exact test. Comparison of binary data were analysed by using logistics regression to calculate odds ratio, 95% confidence interval and p-value. A p-value < 0.05 was considered as statistically significant. Mortality data were analysed for the first admission for each unique patient, since this outcome is limited to a single occurrence per individual.

## Results

In total, 1162 admissions were included consisting of 668 unique patients. Baseline information of the study population and information regarding their hospitalisation is reported in Table 1 (see page 21).

**Table 1:** Patient characteristics

Patient characteristics of all unique patients at first admission (n=668)	
Gender, % female	56
Age, years	70 [63 – 76]
BMI, kg/m^2^	24.5 [21.1 – 28.4]
Smoking status, % never/former/present/unknown	0.4 / 59.9 / 38.2 / 1.5
Lung function	
FEV1, L	1.0 [0.8 – 1.4]
FEV1, %pred.	41 [30 – 57]
FEV1/FVC	40 [32 – 52]
Known with chronic NIV, %	2.8
Known with co-morbidities, %	
cardiac	69
respiratory	43
neurological	24
renal	14
diabetes	20
underweight	11
obesity	19
osteoporosis	17
anxiety/depression	32
anaemia	13
dyslipidaemia	39
Number of co-morbidities per patient	3 [2 – 4]
Number of admission in previous year, median [range]	0 [0 – 8]
Variables at admission (n=1162)	
Origin of exacerbation, %	
infectious	62
non-infectious	12
unknown	27
Arterial blood gas	
pH	7.41 [7.35 – 7.44]
pCO2, kPa	6.1 [5.1 – 7.6]
pO2, kPa	7.8 [6.7 – 9.0]
FiO2, %	21 [21 – 27]
Bicarbonate, mmol/L	28.0 [25.1 – 32.2]
Lab results	
CRP, mg/L	25 [5 – 83]
leukocytes, 10^9/L	11.4 [8.7 – 15.6]
% of cases with eosinophils ≥ 0.3 nL^-1^	16.4%
Duration of hospital stay, days *median [Q1-Q3]*	5 [3 – 8]

**Notes**: Results are presented as median [Q1 – Q3] unless otherwise stated

**Abbreviations**: BMI, body mass index; FEV1, forced expiratory volume in 1 s; FVC, forced vital capacity; NIV, non-invasive ventilation; pCO_2_, partial pressure of carbon dioxide; pO_2_, partial pressure of oxygen; FiO_2_, fraction of inspired oxygen; CRP, C-reactive protein

### Non-invasive ventilation at admission

In 1110 admissions (96%), an arterial blood gas (ABG) analysis was performed at admission. Reasons for not performing an ABG in the remaining 52 admissions were: failure to obtain an ABG (31%), admission via another medical specialty (10%), unknown (52%) or other (7%).

In 267 admissions (23% of all admissions), the inclusion criteria for NIV were met. The respiratory acidosis cleared in 63 admissions after initial treatment with bronchodilators and/or oxygen titration. In 154 of the remaining 204 admissions (76%), NIV was started. In 13 of the 50 admissions (26%) where NIV was indicated but not started, the reason for not starting was in agreement with the guidelines. Among the 895 admissions without an indication for NIV, NIV was initiated in 22 admissions (3%).

**Fig. 1:**
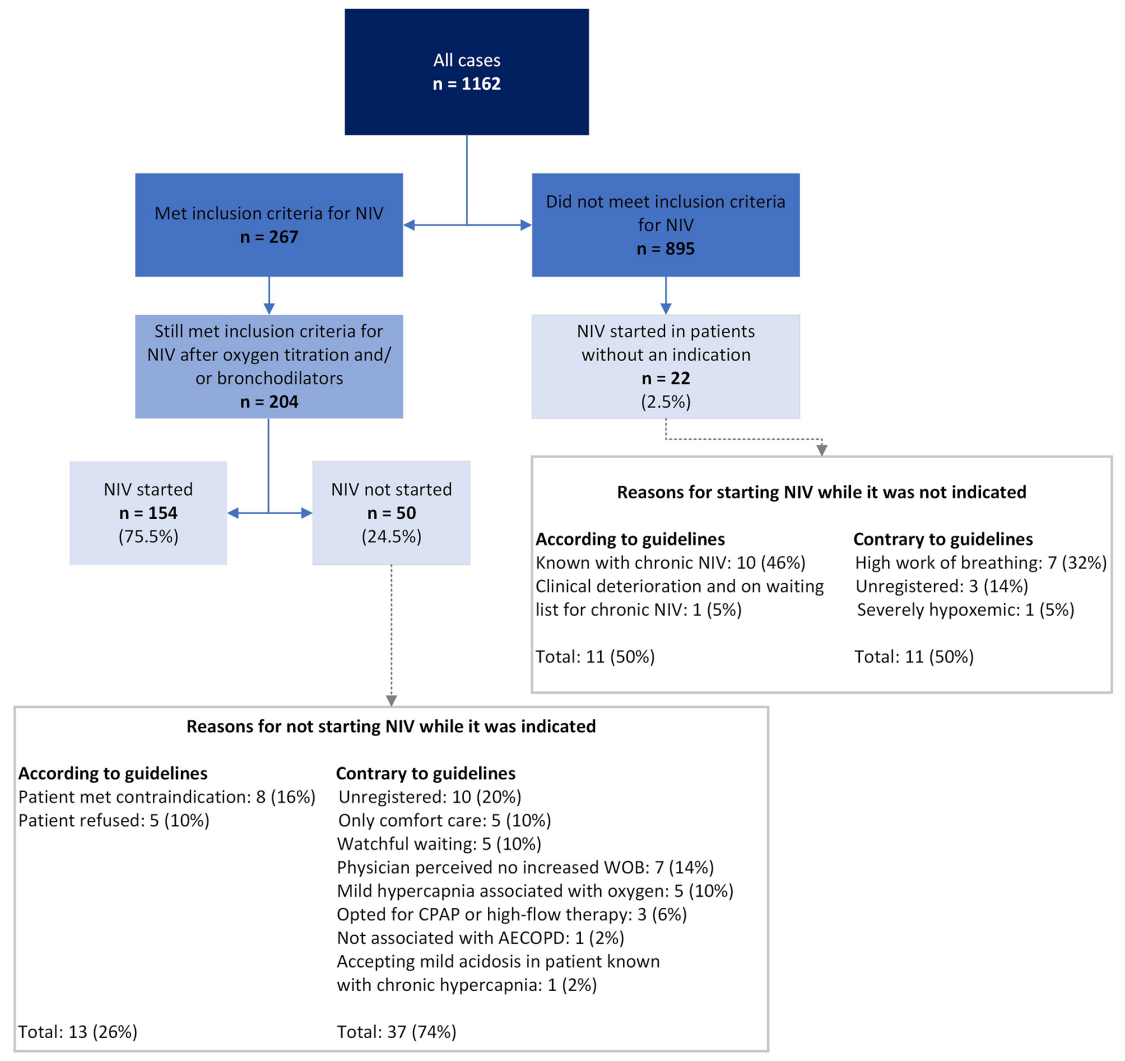
Overview of non-invasive ventilation started at admission. Abbreviations: NIV, non-invasive ventilation; WOB, work of breathing; CPAP, continuous positive airway pressure; AECOPD, acute exacerbation of chronic obstructive pulmonary disease; ABG, arterial blood gas

Figure 1 provides an overview of all admissions and presents the reasons for refraining from NIV when it is indicated and vice versa (all as far as deducible from the retrospective records). Details about NIV treatment at admission can be found in Table 2 (see page 22).

**Table 2:** Treatment details of non-invasive ventilation started if indicated at admission

	All admissions (n=154)	Hospital A (n=43)	Hospital B (n=111)	P-value
Place of treatment, %				0.003
Pulmonary ward	36	58	28	
ICU	42	30	47	
ER	21	12	25	
IPAP, cmH_2_O	15 [13 – 18]	16 [14 – 23]	15 [13 – 18]	0.008
EPAP, cmH_2_O	6 [5 – 8]	6 [5 – 6]	5 [5 – 8]	0.577
NIV length, days	0.9 [0.2 – 2.0]	1.0 [0.5 – 3.0]	0.5 [0.1 – 1.3]	0.007
Reasons for NIV termination, %				0.002
According to protocol	53	40	59	
NA, started/known with chronic NIV	10	26	4	
Patient wanted to terminate	10	9	10	
Palliative trajectory	8	7	8	
NIV was not effective	3	5	3	
Transfer to invasive ventilation	8	5	10	
Mask problems	1	2	-	
Switch to high-flow therapy	3	7	2	
Clinical improvement (still acidotic)	3	-	4	
Unknown	1	-	2	

**Notes**: Results are presented as median [Q1 – Q3] unless otherwise stated

**Abbreviations**: ICU, intensive care unit; ER, emergency room; IPAP, inspiratory positive airway pressure; EPAP, expiratory airway pressure; NIV, non-invasive ventilation; NA, not applicable

### Non-invasive ventilation during hospitalization

**Fig. 2:**
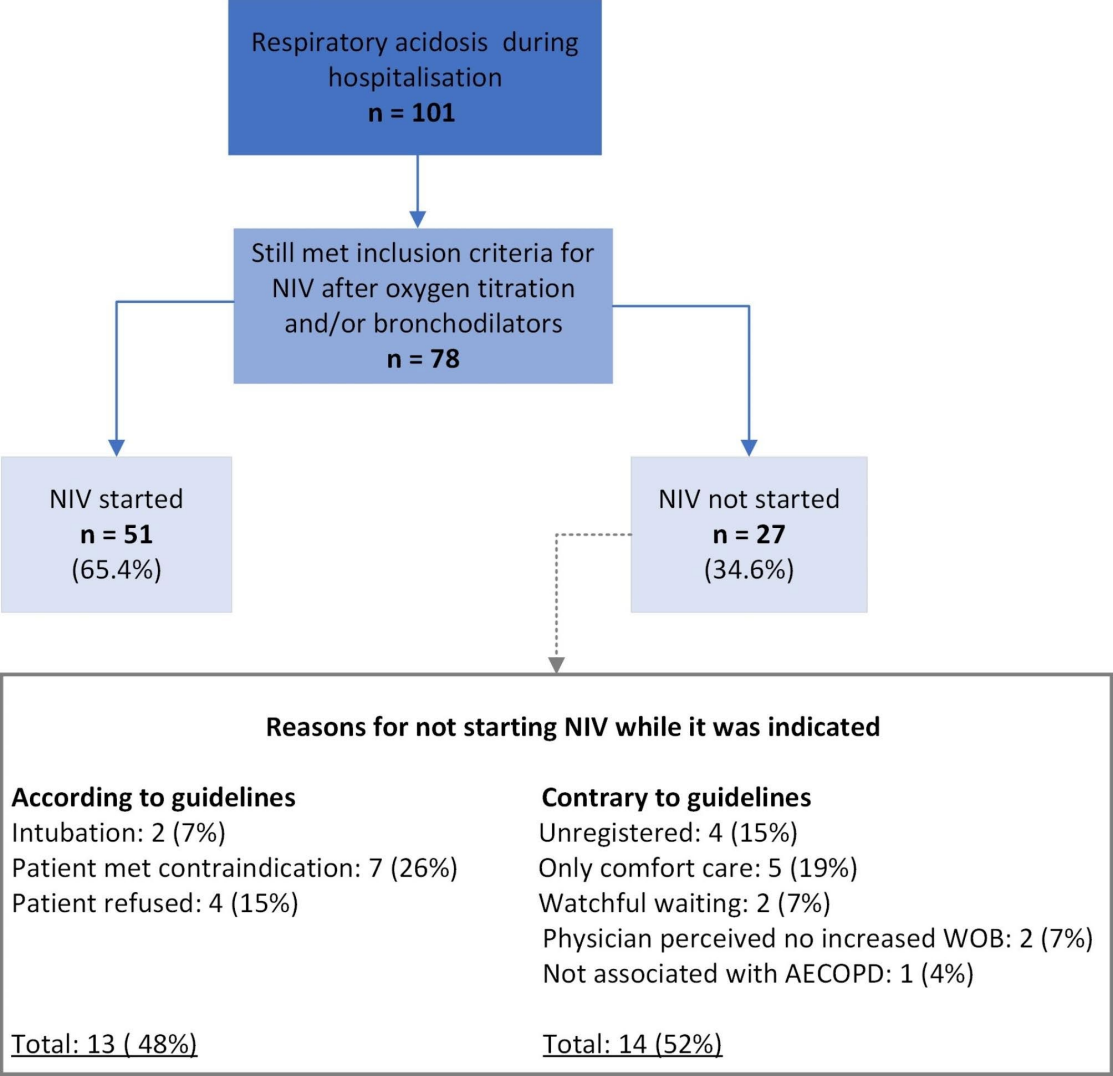
Overview of non-invasive ventilation started later during hospitalisation. Abbreviations: NIV, non-invasive ventilation; WOB, work of breathing; AECOPD, acute exacerbation of chronic obstructive pulmonary disease

In 101 admissions (9% of all admissions), the patient deteriorated during their hospital stay. This includes patients who had an indication for NIV earlier during their admission (n = 25), but irrespective of whether NIV was initiated at admission or not, all these patients achieved an arterial pH within the normal range without NIV before the onset of the deterioration. Of the 101 admissions, 78 met the inclusion criteria for NIV after an alteration in oxygen or bronchodilators. In 51 of the 78 admissions (65%), NIV was initiated. In 13 of the 27 admissions (48%) where NIV was indicated but not started, the reason for not starting was in agreement with the guidelines. Figure 2 shows an overview of admissions where patients later deteriorated and provides the reasons why NIV was not started while it was indicated. Details about the NIV treatment during hospitalization can be found in Table 3 (see page 23).

**Table 3:** Treatment details of non-invasive ventilation started later during hospitalisation

	All admissions (n=51)	Hospital A (n=14)	Hospital B (n=37)	P-value
Place of treatment, %				0.546
Pulmonary ward	57	64	54	
ICU	43	36	46	
IPAP, cmH_2_O	16 [13 – 21]	22 [15 – 25]	14 [12 – 20]	0.012
EPAP, cmH_2_O	6 [ 4 – 8]	6 [6 – 8]	5 [4 – 7.8]	0.021
NIV length, days	1.0 [0.2 – 3.2]	2.0 [1.0 – 4.5]	1.0 [0.2 – 3.0]	0.080
Reasons for NIV termination, %				0.758
According to protocol	39	57	32	
NA, started/known with chronic NIV	4	7	3	
Patient wanted to terminate	12	7	14	
Palliative trajectory	16	7	19	
NIV was not effective	-	-	-	
Transfer to invasive ventilation	22	21	22	
Mask problems	2	-	3	
Switch to high-flow therapy	4	-	5	
Clinical improvement (still acidotic)	2	-	3	
Unknown	-	-	-	

**Notes**: Results are presented as median [Q1 – Q3] unless otherwise stated

**Abbreviations**: ICU, intensive care unit; IPAP, inspiratory positive airway pressure; EPAP, expiratory airway pressure; NIV, non-invasive ventilation; NA, not applicable

### Mortality

The overall in-hospital and 90-day mortality rate for the cohort of 668 unique patients were 6% and 14%, respectively. In patients with an indication for NIV at admission, mortality at 90 days was significantly lower in patients who received NIV compared to patients who did not receive NIV due to reasons not in agreement with the guidelines (Table 4). The in-hospital mortality was not significantly different between these groups. In patients with an indication for NIV later during hospitalisation, no significant differences in mortality rates were seen between who received NIV and patients who did not receive NIV due to reasons not in agreement with the guidelines.

**Table 4:** Comparison of patient characteristics and mortality in patients who had an indication for non-invasive ventilation

	Unique patients with an indication for NIV at admission			Unique patients with an indication for NIV later during hospitalisation		
	NIV initiated (n = 78)	NIV not initiated for reasons contrary to guidelines (n= 21)	p-value	NIV initiated (n=28)	NIV not initiated for reasons contrary to guidelines (n=6)	p-value
**Patient characteristics**						
Gender, % female	59.0	57.1	1.000	57.1	83.3	0.370
Age, years	69.0 [63.0 – 73.3]	70.0 [58.0 – 75.0]	0.827	71.0 [67.3 – 79.0]	73.5 [59.0 – 80.0]	0.912
BMI, kg/m^2^	24.2 [20.4 – 27.9]	24.1 [21.0 – 27.9]	0.955	24.7 [21.6 – 28.0]	22.9 [19.4 – 25.8]	0.439
FEV1, %pred.	31.5 [23.7 – 39.4]	33.7 [21.4 – 41.4]	0.808	36.1 [27.9 – 41.7]	33.1 [23.4 – NA]	0.761
Known with co-morbidities, %						
cardiac	68.8	85.7	0.171	57.1	50.0	1.000
respiratory	39.0	38.1	1.000	21.4	0.0	0.562
Arterial blood gas at moment of NIV indication						
pH pCO2, kPa pO2, kPa	7.27 [7.23 – 7.31] 9.7 [8.3 – 10.9] 8.4 [6.5 – 9.9]	7.33 [7.28 – 7.34] 8.3 [6.6 – 8.7] 8.2 [6.7 – 11.7]	<0.001 0.002 0.821	7.28 [7.25 – 7.31] 9.3 [7.9 - 10.3] 8.5 [6.6 – 10.6]	7.27 [7.23 – 7.30] 10.1 [8.3 – 10.9] 6.7 [5.9 – 10.2]	0.741 0.644 0.238
CRP, mg/L	31.5 [6.8 – 89.5]	32.0 [4.0 – 147.0]	0.830	30.5 [4.3 – 133.8]	34.5 [4.0 – 103.8]	0.878
**Mortality**						
In-hospital mortality, %	14.1	23.8	0.289 (OR 0.525 [0.160-1.726])	21.4	50.0	0.166 (OR 0.273 [0.043 – 1.713])
Mortality at 90 days, %	24.7	47.6	0.046 (OR 0.360 [0.132-0.980])	46.4	83.3	0.131 (OR 0.173 [0.018 – 1.681])

**Notes**: Only unique patients (first admission) are included in the analysis. Results are presented as median [Q1 – Q3] unless otherwise stated. For the mortality data, the odds ratio with 95%CI are included

**Abbreviations**: BMI, body mass index; FEV1, forced expiratory volume in 1 s; NIV, non-invasive ventilation; pCO_2_, partial pressure of carbon dioxide; pO_2_, partial pressure of oxygen; CRP, C-reactive protein; NA, not available (due to missing values)

Furthermore, Table 4 shows that patients who were initiated on NIV at admission had a more severe hypercapnic acidosis than patients who did not receive NIV.

### Variation in NIV treatment between centers

No difference in the initiation of NIV among indicated patients was observed between the centers, both when NIV was initiated at admission (hospital A vs. B: 78.2 vs. 74.5%, p = 0.588, OR 0.815 [0.390–1.706]) and later during hospitalisation (hospital A vs. B: 60.9 vs. 67.3%, p = 0.588, OR 1.321 [0.482–3.625]). Table 2 provides details on NIV treatment administered upon admission at both centers, showing higher inspiratory positive airway pressure (IPAP) levels and longer treatment duration in hospital A compared to hospital B. Furthermore, a significant difference was seen in the place of NIV treatment and the reasons for NIV termination between both centers. No differences were found between the two centers for in-hospital mortality (hospital A vs. B: 18.2 vs. 11.1%, p = 0.380, OR 0.563 [0.156–2.030]) and 90-day mortality (hospital A vs. B: 24.2 vs. 25.0%, p = 0.939, OR 1.042 [0.365–2.972]) in patients where NIV was initiated at admission. Table 3 contains information on NIV treatment initiated later during hospitalization at both centers, revealing lower NIV pressures were given in hospital B compared to hospital A. No differences were found between the two centers for in-hospital mortality (hospital A vs. B: 10.0 vs. 27.8%, p = 0.292, OR 3.461 [0.344–34.843] and 90-day mortality (hospital A vs. B: 50.0 vs. 44.4%, p = 0.778, OR 0.800 [0.170–3.767]) in patients were NIV was initiated later during hospitalisation. Additional information regarding patient characteristics of all patients who received NIV, specified per hospital, can be found in Table S1 of the Supplementary Materials.

## Discussion

This is the first study describing the use of NIV for acute COPD exacerbations in two medical centers in the Netherlands. Our findings demonstrate that NIV was initiated in 76% of the admissions where NIV was indicated upon admission, while this was the case in 65% of the admissions where the patient deteriorated later during hospitalization.

Previous studies evaluating the adherence to NIV guidelines at admission in clinical practice outside the Netherlands have shown variable results, with guideline rates ranging from 24 to 74% [7–11]. One prior survey study was performed in the Netherlands and showed a guideline adherence rate of 65% [12]. Our results showed that NIV was initiated in 76% of the admissions where NIV was indicated patients at admission. If we take into account patients who were not initiated on NIV due to reasons in line with the guidelines, the adherence rate concerning NIV initiation at admission reached 82%, indicating a relatively high level of adherence to guidelines. Vice versa, our findings show that the initiation of NIV in patients without an indication is infrequent. In only 2.5% of the admissions where NIV was not indicated, NIV was started. Out of those admissions, only half received NIV against the guidelines. These outcomes are superior compared to previous studies [7, 8]. Additionally, in terms of obtaining an ABG at admission, our findings exceed reported rates in literature [7, 8, 10, 11, 13–15]. A possible explanation for these higher adherence rates might be that our study was performed in two large centers with clear protocols and considerable experience in the field of NIV. Furthermore, the availability of well-educated staff and NIV facilities may play a role.

To our knowledge, only one study [7] investigated the adherence to NIV guidelines when patients deteriorated later on during hospitalisation. They included patients who were admitted with a normal pH and developed an acidosis later during hospitalisation and they showed an adherence rate of 47% in this group. Again, our results show a higher level of adherence to guidelines. Our study demonstrated that NIV was initiated in 65% of the admissions where patients had a NIV indication later during hospitalisation. Considering patients who were not given NIV because of a contra-indication or because patients refused, the adherence rate in patients who deteriorated later on during hospitalisation reaches 82%, matching the adherence rate observed at admission. In patients who deteriorated later, the rationale for the non-initiation of NIV appears to be better described in the medical records, implying that the medical staff is more aware of the indication and possible need for NIV during hospitalisation than at admission.

The mortality rates in both the overall population and specifically in the group of patients who were treated with NIV are consistent with those reported in prior research on acute exacerbations of COPD [13, 16–19]. In patients with an indication for NIV at admission, the group of patients who did not receive NIV due to reasons not according to the guidelines had a worse survival compared to those who received NIV. This is noteworthy because these patients had a less severe respiratory acidosis, which is expected to correspond to lower mortality rates. These findings may emphasize the importance of NIV initiation when indicated. In addition, the absolute mortality rates were higher in patients who later deteriorated during hospitalisation compared to those who required NIV upon admission. It appears that patients who later deteriorated have worse outcomes and that these patients have less benefit of NIV compared to patients with an NIV indication at admission, as also previously reported in literature [7, 20]. This worse outcome is also reflected in the large number of patients who were initiated on NIV during hospitalisation but terminated NIV due to the transfer to invasive ventilation or to receive comfort care only.

While no disparities in NIV initiation were observed between centers, notable differences were identified in the NIV treatment between both participating centers. The variation in NIV duration started at admission can be clarified by the difference in protocols between both hospitals in the manner of weaning from NIV. Hospital A employs a stepwise reduction by gradually reducing the number of hours per day on the ventilator while hospital B immediately withdraws NIV once the respiratory acidosis has been resolved. Since mortality rates were not affected, these findings support earlier reported results that a stepwise reduction of NIV is equally effective compared to an immediate withdrawal [21, 22]. Next to the length of NIV treatment, the most noticeable differences between the two centers revolved around the place of NIV treatment and the reasons for NIV termination. Notably, hospital A treated patients more frequently on the pulmonary ward rather than in the ICU, which can be possibly attributed to logistical variations between the centers. Additionally, the difference in reasons for NIV termination is primarily explained by the fact that hospital A initiated more patients on chronic NIV than hospital B. This observation seems logical since hospital A is an academic hospital with a specialized expertise in chronic ventilation. Despite these disparities in NIV treatment practices, the mortality rates between the centers did not show any significant differences.

Although this study shows a relatively good adherence to NIV guidelines, there is still potential for enhancing NIV treatment in clinical practice. This is evident from the subset of patients who met the criteria for NIV treatment but did not receive it due to reasons contrary to guidelines. The most benefit can probably be gained by improving the adherence to NIV guidelines at admission, since nearly three-quarters of patients who met the criteria for NIV treatment but did not receive it at admission, did not have a justifiable reason for its non-initiation. In a large portion of these patients, the reason was not registered or the possibility of NIV was not mentioned in the medical records. This may indicate unawareness among healthcare providers at the emergency department about the indication and benefit of NIV in acute COPD exacerbations, and could be improved with more training. Furthermore, healthcare providers seem to be reluctant to initiate NIV when indicated, as suggested by several reasons provided for non-initiation such as watchful waiting and no increased work of breathing. In order to improve NIV use in clinical practice, further research should focus on training and on investigating why healthcare providers are reluctant to use NIV in clinical practice.

This study has several limitations. First of all, this study was performed in only two centres, which challenges the extrapolation of the results to the general population of hospitalized COPD patients, especially when considering the potential variability between centers even in the same country [13, 23]. On the other hand, both an academic and a non-university center were included. Second, due to its retrospective design, the study is limited by some degree of missing data, for example reasons why NIV was not started despite being indicated. It is plausible that NIV treatment was considered but not initiated due to justifiable reasons, but that this rationale was not documented in the medical records. And last, the classification of patients who did not receive NIV when it was indicated into two categories (on reasons in agreement with and contrary to the guidelines) may be open to debate. Especially, the rationale ‘to only start comfort care’ could be placed into either one of those categories, depending on local protocols. Since our local NIV protocol does not state comfort care as a contraindication for NIV initiation, we categorized it as a reason contrary to guidelines. Another justification for placing it in that category is that previous research has demonstrated the effectiveness of NIV in alleviating dyspnoea in end-stage disease patients [24, 25].

In conclusion, we showed a good adherence to NIV guidelines during acute exacerbations of COPD in two medical centers in the Netherlands. Failure to initiate NIV when indicated at admission may have a detrimental effect on patient outcomes, highlighting the importance of NIV initiation in such cases.

### Electronic supplementary material

Below is the link to the electronic supplementary material.

**Additional file 1**: Table S1. Description of data: Patient characteristics of patients who were initiated on NIV for acute respiratory failure, specified per center.

**Additional file 2**: Non-invasive ventilation protocol. Protocol for non-invasive ventilation in patients with acute respiratory insufficiency due to a COPD exacerbation.

## Data Availability

The datasets used and/or analysed during the current study are available from the corresponding author on reasonable request.

## List of abbreviations

ABG: arterial blood gas
BMI: body mass index
COPD: chronic obstructive pulmonary disease
CRP: C-reactive protein
EPAP: expiratory airway pressure
ER: emergency room
FEV1: forced expiratory volume in 1 s
FVC: forced vital capacity
ICU: intensive care unit
IPAP: inspiratory positive airway
NA: not applicable
NIV: non-invasive ventilation
PaCO_2_: partial pressure of carbon dioxide
PaO_2_: partial pressure of oxygen
