# Supplementary material for: Clinical practice of non-invasive ventilation for acute exacerbations of chronic obstructive pulmonary disease
Source: Respir Res. 2023 Aug 23;24:208. doi: 10.1186/s12931-023-02507-1 (PMC10464197; doi:10.1186/s12931-023-02507-1)
Supplement: Supplementary file 1 — Additional file 1: Table S1. Description of data: Patient characteristics of patients who were initiated on NIV for acute respiratory failure, specified per center. [file 12931_2023_2507_MOESM1_ESM.docx]

**Table S1** Patient characteristics of patients who were initiated on NIV for acute respiratory failure, specified per center

|  | Patients initiated on NIV  at admission | | | Patients initiated on NIV  later during hospitalization | | |
| --- | --- | --- | --- | --- | --- | --- |
|  | Hospital A (n=43) | Hospital B (n=111) | p-value | Hospital A (n=14) | Hospital B  (n=37) | p-value |
| Gender, % female | 46.5 | 57.7 | 0.279 | 64.3 | 73.0 | 0.732 |
| Age, years | 68.8 (7.7) | 66.6 (8.4) | 0.144 | 67.9 (5.7) | 71.9 (8.4) | 0.061 |
| BMI, kg/m^2^ | 24.5 (6.47) | 25.0 (6.2) | 0.339 | 22.45 (3.7) | 25.8 (5.6) | 0.043 |
| FEV1, %pred. | 30.0 (9.4) | 33.6 (11.0) | 0.067 | 31.2 (16.6) | 42.0 (13.0) | 0.019 |
| Known with co-morbidities, %  cardiac  respiratory | 79.1 48.8 | 66.4 40.9 | 0.169 0.468 | 64.3 14.3 | 59.5 37.8 | 1.000 0.176 |
| Arterial blood gas at moment of NIV indication  pH, *median [Q1 – Q3]*  pCO2, kPa  pO2, kPa,  *median [Q1 – Q3]* | 7.29 [7.24 – 7.32] 9.8 (2.0) 8.4 [7.1 – 10.8] | 7.26 [7.22 – 7.30] 10.1 (2.4) 8.0 [6.1 – 9.3] | 0.155 0.532 0.184 | 7.30 [7.26 – 7.31] 9.7 [7.8 – 11.8] 8.1 [7.1 – 12.2] | 7.29 [7.26 – 7.33] 9.2 [8.1 – 10.4] 8.4 [6.3 – 9.8] | 0.815 0.520 0.230 |
| CRP, mg/L,  *median [Q1 – Q3]* | 38 [9 – 134] | 21 [6 – 64] | 0.173 | 32 [4 – 53] | 32 [6 – 133] | 0.180 |

***Notes****: Results are presented as mean (SD) unless otherwise stated.****Abbreviations****: BMI, body mass index; FEV1, forced expiratory volume in 1 second; NIV, non-invasive ventilation; pCO_2_, partial pressure of carbon dioxide; pO_2_, partial pressure of oxygen; CRP, C-reactive protein*
