# Supplementary figures and images for: Clinical practice of non-invasive ventilation for acute exacerbations of chronic obstructive pulmonary disease
Source: Respir Res. 2023 Aug 23;24:208. doi: 10.1186/s12931-023-02507-1 (PMC10464197; doi:10.1186/s12931-023-02507-1)

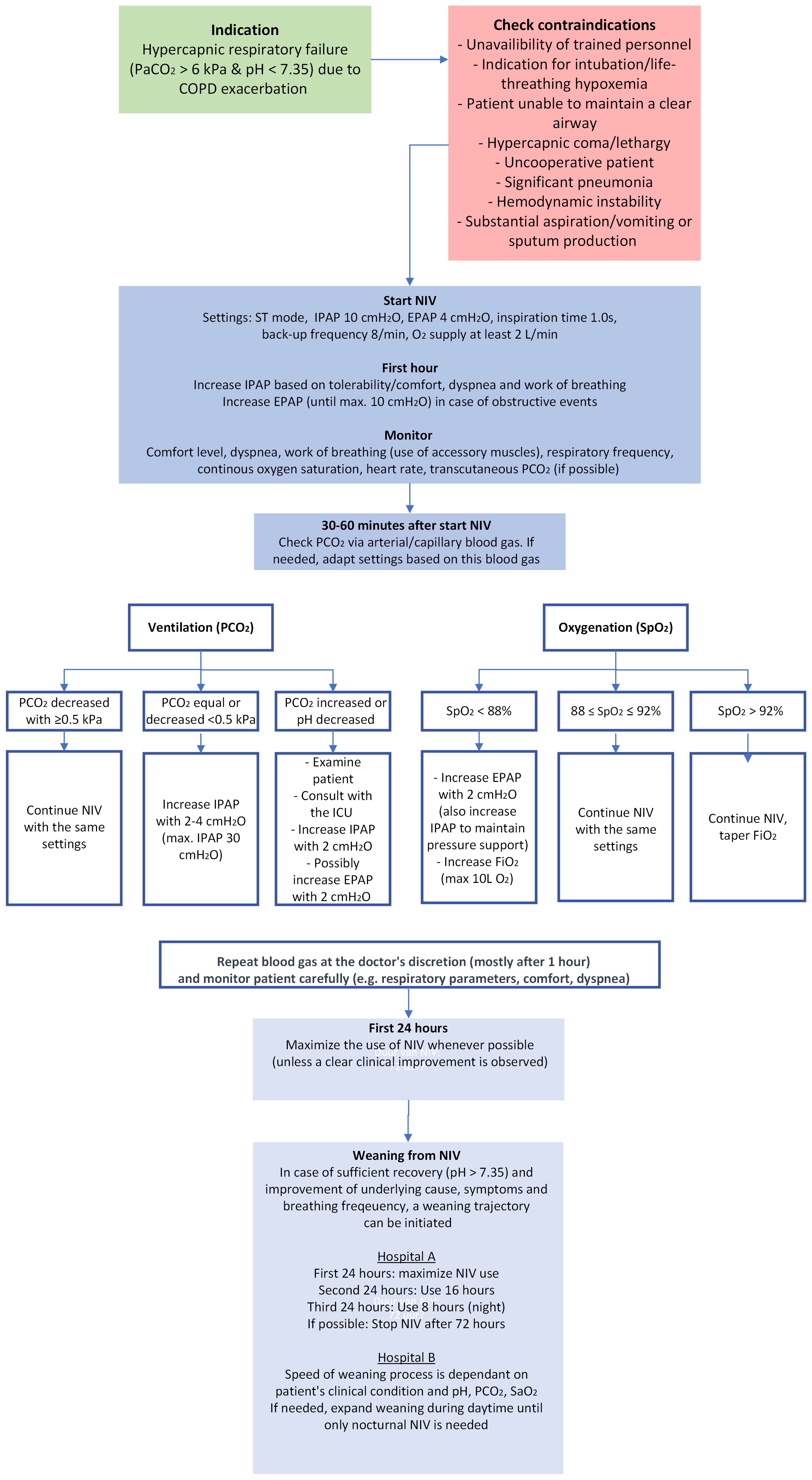

Supplement: Supplementary file 2 — Additional file 2: Non-invasive ventilation protocol. Protocol for non-invasive ventilation in patients with acute respiratory insufficiency due to a COPD exacerbation. [file 12931_2023_2507_MOESM2_ESM.jpeg]
